# Ki67, chemotherapy response, and prognosis in breast cancer patients receiving neoadjuvant treatment

**DOI:** 10.1186/1471-2407-11-486

**Published:** 2011-11-14

**Authors:** Peter A Fasching, Katharina Heusinger, Lothar Haeberle, Melitta Niklos, Alexander Hein, Christian M Bayer, Claudia Rauh, Ruediger Schulz-Wendtland, Mayada R Bani, Michael Schrauder, Laura Kahmann, Michael P Lux, Johanna D Strehl, Arndt Hartmann, Arno Dimmler, Matthias W Beckmann, David L Wachter

**Affiliations:** 1 Department of Medicine, Division of Hematology and Oncology, David Geffen School of Medicine, University of California at Los Angeles, California, USA; 2 Department of Gynecology and Obstetrics, Erlangen University Hospital, Erlangen, Germany; 3 Institute of Diagnostic Radiology, Erlangen University Hospital, Erlangen, Germany; 4 Institute of Pathology, Erlangen University Hospital, Erlangen, Germany; 5 Institute of Pathology, St Vincentius Hospital, Karlsruhe, Germany

## Abstract

**Background:**

The pathological complete response (pCR) after neoadjuvant chemotherapy is a surrogate marker for a favorable prognosis in breast cancer patients. Factors capable of predicting a pCR, such as the proliferation marker Ki67, may therefore help improve our understanding of the drug response and its effect on the prognosis. This study investigated the predictive and prognostic value of Ki67 in patients with invasive breast cancer receiving neoadjuvant treatment for breast cancer.

**Methods:**

Ki67 was stained routinely from core biopsies in 552 patients directly after the fixation and embedding process. HER2/neu, estrogen and progesterone receptors, and grading were also assessed before treatment. These data were used to construct univariate and multivariate models for predicting pCR and prognosis. The tumors were also classified by molecular phenotype to identify subgroups in which predicting pCR and prognosis with Ki67 might be feasible.

**Results:**

Using a cut-off value of > 13% positively stained cancer cells, Ki67 was found to be an independent predictor for pCR (OR 3.5; 95% CI, 1.4, 10.1) and for overall survival (HR 8.1; 95% CI, 3.3 to 20.4) and distant disease-free survival (HR 3.2; 95% CI, 1.8 to 5.9). The mean Ki67 value was 50.6 ± 23.4% in patients with pCR. Patients without a pCR had an average of 26.7 ± 22.9% positively stained cancer cells.

**Conclusions:**

Ki67 has predictive and prognostic value and is a feasible marker for clinical practice. It independently improved the prediction of treatment response and prognosis in a group of breast cancer patients receiving neoadjuvant treatment. As mean Ki67 values in patients with a pCR were very high, cut-off values in a high range above which the prognosis may be better than in patients with lower Ki67 values may be hypothesized. Larger studies will be needed in order to investigate these findings further.

## Background

The aim in modern, individualized medicine is to identify patients who have an unfavorable prognosis -- or even better, to identify patients who may be capable of benefiting from an improved prognosis associated with a specific form of treatment. There has recently been discussion on whether the proliferation marker Ki67 might be suitable for inclusion in everyday clinical practice, although it was considered that the marker is not yet ready for routine use [1]. In novel multigene tests, however, proliferation has a major impact on calculations of the risk of recurrence. Ki67 itself is already part of a multigene test [2] that is being used in clinical studies such as the TailorX and the planB studies [3]. In a study including a group of women receiving antihormonal treatment, it has been suggested that using Ki67, estrogen receptor (ER), progesterone receptor (PR), and the HER2/neu receptor (HER2) may have a prognostic value similar to that of a multigene prognostic score [4].

The correlation of Ki67 with breast cancer outcome has been demonstrated both in patients undergoing chemotherapy and in patients treated with antihormonal therapy [5], and some of its effect on the outcome appears to be unrelated to any specific form of therapy. It might therefore be reasonable to assume that the correlation of Ki67 with breast cancer outcome involves a mixture of prognostic and predictive effects.

The neoadjuvant setting is a useful model for investigating the value of Ki67 as a predictive and prognostic factor. Some neoadjuvant studies have investigated Ki67 in relation to complete pathological response (pCR), and one has examined its association with disease progression during neoadjuvant therapy [6]. The majority of studies investigating pCR have identified a high Ki67 proliferation rate as a predictive factor for a higher rate of pCR (reviewed in [5]), but the only study that has examined progression during neoadjuvant chemotherapy found that patients in whom progression occurred had a higher proliferation rate than those who responded to chemotherapy [6]. This suggests a nonlinear effect of Ki67 on the treatment response and possibly the prognosis.

As a variety of studies have now established that pCR is a surrogate marker for prognosis [7-9], a cohort capable of providing pretreatment predictive markers, information on pCR, and follow-up data might be able to identify patients who still have a favorable prognosis despite a lack of complete response to neoadjuvant therapy -- while vice versa, it might also help identify patients who still have a poorer prognosis even after a pCR.

The aim of the present study was therefore to investigate Ki67 immunohistochemistry with regard to its ability to predict treatment response in a group of neoadjuvantly treated breast cancer patients and to correlate Ki67 expression with prognosis within the different response groups to chemotherapy.

## Methods

### Patients

**Figure 1:**
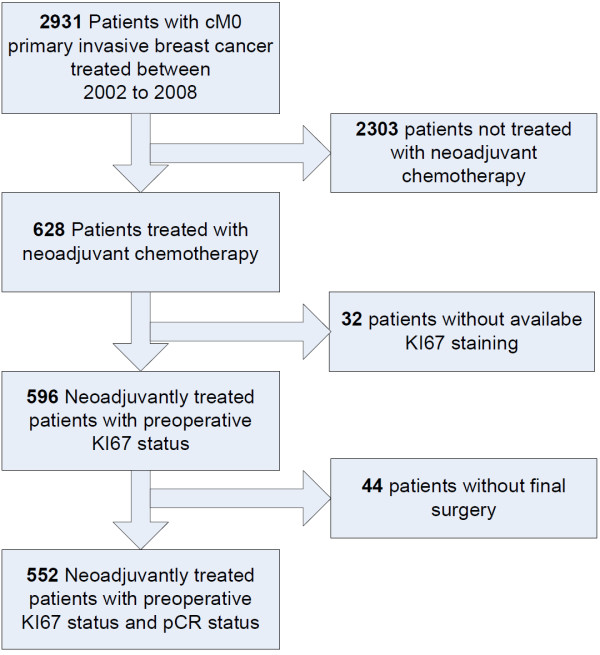
**Patient selection**.

The patients included in this retrospective study were selected from all patients with invasive breast cancer treated with neoadjuvant chemotherapy at the University Breast Center for Franconia (Bavaria, Germany) between January 2002 and December 2008. For inclusion in the study, the patients had to be at least 18 years of age and had to have undergone surgery following the neoadjuvant chemotherapy. Information on the following parameters had to be available from the pretreatment assessment: patient's age, tumor size, estrogen receptor status, progesterone status, HER2 status, grading, and proliferation status as assessed by Ki67 staining. The patient selection process is shown in Figure 1.

Approval for the analyses conducted in the study was received from the ethics committee of the medical faculty at the University of Erlangen-Nuremberg. Informed consent was obtained from patients whose data have been included in this study.

### Clinical data

The University Breast Center for Franconia has received certification from the German Cancer Society and the German Society for the Study of Breast Diseases (*Deutsche Gesellschaft für Senologie*). To obtain certification, a breast center has to document each case of breast cancer, including patient and tumor characteristics, treatment data, and some epidemiological data (http://www.onkozert.de). As part of the certification process, it is checked whether treatment decisions for patients are in accordance with the German guidelines for the treatment of breast cancer. Follow-up information regarding local recurrences, distant metastases, and death has to be provided for up to 10 years after the initial diagnosis. All histopathological data also have to be documented from the original pathological reports, including tumor size, axillary lymph-node status, grading, and estrogen receptor, progesterone receptor, and HER2/neu status. Breast centers and their data quality are audited annually as part of the continuous certification process. Data obtained through these processes were used in the analysis presented here. A complete pathological response was defined as no evidence of any tumor cells in the breast and no evidence of any tumor cells in the axilla.

### Histopathological data and pCR assessment

All of the histopathological information used in the analysis was directly documented from the original pathology reports, which were reviewed by two investigators. Grading, tumor type, estrogen receptor status, progesterone receptor status, HER2/neu status, and proliferation status as assessed by Ki67 staining have been routinely recorded at the breast center since 1995 and performed on formalin-fixed, paraffin-embedded tumor tissue. Monoclonal mouse antibodies against estrogen receptor-alpha (clone 1D5; 1: 200 dilution, DAKO, Denmark), monoclonal mouse antibody against the progesterone receptor (clone pgR636, 1: 200 dilution, DAKO, Denmark), and monoclonal antibody against Ki67 (clone MIB-1, 1: 200 dilution, DAKO, Denmark) were used to stain the pretreatment core biopsies. The percentage of positively stained cells was included in the pathology reports. The tumors were considered to be positive for the estrogen and progesterone receptors if 10% or more of the cells showed positive staining. The cut-off point for Ki67 status was regarded as more than 13% positively stained cells, in accordance with the biological analysis presented by Cheang et al. [10]. This cut-off was chosen because it lies in the range reported in published studies [5] and also has a correlate with molecular subtypes of breast cancer [10]. A polyclonal antibody against HER2/neu (1: 200 dilution, DAKO, Denmark) was used, and HER2 status was given in the pathology reports as negative, 0, 1+, 2+, or 3+ in accordance with the guidelines published by Sauter et al. [11]. Tumors with a score of 0 or 1+ were regarded as HER2-negative and those with a score of 3+ were regarded as HER2-positive. Tumors with a 2+ staining were tested for gene copy numbers of Her2 by chromogene in-situ hybridization. Using a kit with two probes of different colors (ZytoDot, 2C SPEC HER2/CEN17, Zyto Vision Ltd., Bremerhaven, Germany), the gene copy numbers of HER2 and centromeres of the corresponding chromosome 17 were retrieved. A HER2/CEN17 ratio of ≥ 2.2 was considered as amplification of HER2.

Scoring was carried out in a standardized way by a group of dedicated pathologists in routine surgical pathology. With regard to Ki67, areas with the highest Ki67 labeling were investigated and approximately 500 cells were counted with 400-fold magnification.

The pCR assessment was based on histopathological reports, all from one institution. Patients with an ypT0 ypN0 assessment were considered to have achieved a pathological complete response (pCR). No invasive or noninvasive residual tissue in the breast or nodes was allowed, as in Sinn's assessment [12].

### Statistical considerations

The characteristics of patients with pCRs and patients without pCRs were compared using the appropriate unpaired statistical tests. Welch's *t*-tests were used for continuous characteristics, chi-squared tests with continuity correction for categorical characteristics, and the Armitage trend test for ordinal categorical characteristics.

For each risk parameter, the odds ratio (OR) for pCR versus no pCR was calculated using simple logistic regression models. To study the additional predictive value of Ki67 relative to pCR, a multiple logistic regression model including all risk factors except for Ki67 was fitted. Backward stepwise variable selection was carried out to obtain the best model in accordance with the Akaike information criterion (the final model). The risk factor of Ki67 was then added to the final model (providing the extended final model) and the two models were compared both with the likelihood ratio test and with its receiver operating characteristic (ROC) curves. In addition, the predictive power of the extended final model was measured using the area under the curve (AUC) and the sensitivity and specificity of the optimal cut-off point were assessed in accordance with Youden's index. To take overfitting into account, these predictive measures were evaluated using 20-fold cross-validation and with the 0.632+ bootstrapping method with 500 bootstrap samples [13,14].

Overall survival, distant disease-free survival, and local recurrence-free survival were the outcomes of interest in the prognosis analyses. Survival rates were estimated using the Kaplan-Meier product limit method. Differences between survival curves were tested using the logrank test. Multiple Cox proportional hazard (PH) models were used to obtain hazard ratios (HRs). For each outcome, a full model containing all risk factors except for pCR was constructed, and the final model was again contained using backward stepwise variable selection. The extended final model with the remaining risk factors and also pCR was fitted. The final model and the extended final model were compared using the likelihood ratio test to assess the prognostic value of pCR. The proportional hazard assumptions were checked using tests that correlate scaled Schoenfeld residuals with a suitable time transformation [15].

An optimal cut-off point for Ki67 was calculated with respect to pCR status, using the minimum *P *value approach. Within the 10th and 90th percentiles of Ki67, all values for this variable are considered as potential cut-off points. The optimal cut-off point is then taken in such a way that the *P *value of the simple logistic regression model, with pCR as the target variable and Ki67 categories (below and above the cut-off point, respectively) as predictor variables, is a minimum.

All of the tests were two-sided, and a *P *value < 0.05 was regarded as statistically significant. Calculations were carried out using the R system for statistical computing (version 2.11.1; R Development Core Team, Vienna, Austria, 2010). Functions from the Daim library were used for bootstrapping.

## Results

### Patient characteristics

A total of 552 patients (mean age 53.3 ± 11.8 years) were included in the study. Their mean body mass index (BMI) was 26.1 ± 5.1. Before the chemotherapy treatment, most of the patients had cT2 tumors 2-5 cm in size (67.5%) and cT1 tumors up to 2 cm in size (16.7%). A relevant proportion of the patients had cT4 cancers (11.1%). With regard to the other characteristics, ductal tumors (80.8%) and a grading of 1 or 2 (65.7%) were the most commonly observed categories. With a cut-off value of > 13% positively stained tumor cells for Ki67, most of the tumors (70.7%) were classified as having a high level of Ki67 proliferation. The patients' characteristics are summarized in Table 1. The median follow-up time was 2.8 years, with 68 deaths, 78 distant metastases, and 31 local recurrences.

**Table 1:** Pretreatment patient characteristics and univariate associations with pathological complete remission

	All		pCR no		pCR yes		
	
	Mean or n	SD or %	Mean or n	SD or %	Mean or n	SD or %	*P*
Age	53.3	11.8	54.2	11.5	50.0	12.3	< 0.01
BMI	26.1	5.1	26.4	5.3	25.1	4.1	< 0.01
cT							
1	92	16.7	60	65.2	32	34.8	< 0.0001
2	372	67.5	292	78.5	80	21.5	
3	26	4.7	24	92.3	2	7.7	
4	61	11.1	56	91.8	5	8.2	
Grading							
1	28	5.6	26	92.9	2	7.1	< 0.00001
2	301	60.1	269	89.4	32	10.6	
3	172	34.3	94	54.7	78	45.3	
Histology							
Ductal	445	80.8	338	76.0	107	24.0	0.001
Lobular	80	14.5	76	95.0	4	5.0	
Other	26	4.7	18	69.2	8	30.8	
ER Status							
Negative	198	35.9	103	52.0	95	48.0	< 0.00001
Positive	354	64.1	329	92.9	25	7.1	
PR Status							
Negative	259	46.9	157	60.6	102	39.4	< 0.00001
Positive	293	53.1	275	93.9	18	6.1	
HER2 Status							
Negative	445	81.4	366	82.2	79	17.8	< 0.00001
Positive	102	18.6	61	59.8	41	40.2	
Ki67							
Low	162	29.3	155	95.7	7	4.3	< 0.00001
High	390	70.7	277	71.0	113	29.0	
							
Postoperative radiation							
No	63	15.9	47	74.6	16	25.4	0.94
Yes	333	84.1	255	74.2	86	25.8	

BMI, body mass index; cT, clinical tumor stage (TNM classification); Ki67, proliferation status, with an immunohistological staining cut-off value of > 13%; pCR, pathological complete remission; ER, estrogen receptor; PR, progesterone receptor.

With regard to the treatments administered, 122 patients (22%) received an anthracycline-based regimen without taxanes; 328 (60%) were treated with anthracyclines and taxanes; and 102 (18%) underwent other treatments (taxane only; anthracycline only; and cyclophosphamide, methotrexate, or fluorouracil only). Fifty of the 103 HER2-positive patients (49%) were treated with trastuzumab in a combined therapy together with an anthracycline- and taxane-based regimen. Among the 52 patients who did not receive neoadjuvant trastuzumab treatment, 25 (48%) received trastuzumab as an adjuvant treatment. The rest of the HER2-positive patients did not receive trastuzumab. There were no associations between the choice of therapy and the most common treatment groups, either with regard to anthracycline treatment (*P *= 0.41) or taxane treatment (*P *= 0.37) in the total group of patients or with regard to trastuzumab treatment (*P *= 0.71) in the group of HER2-positive patients.

### Univariate analysis for the association with pCR

Complete remissions -- i.e., no tumor cells found in the breast and no tumor cells found in the axilla -- were observed in 120 of the 552 patients (21.7%). The common prognostic factors in these patients showed expected associations with pCR. Patients with a pCR were more likely to be younger (50.0 vs. 54.2 y) and were more likely to have a lower BMI (25.1 vs. 26.4 kg/m^2^). Small tumors were associated with a higher pCR rate, as were tumors that were higher-grade, non-lobular, hormone receptor-negative, and HER2-positive.

Using a cut off value of > 13% positively stained cancer cells, pCRs were observed in 113 of 390 patients (29%) with a high proliferative status as assessed by Ki67 and in seven of 162 patients (4.3%) with a low proliferative status (Table 1).

### Multivariate analysis for predicting pCR

As Ki67 was strongly associated with pCR in the univariate analysis, two multivariate models were constructed in order to investigate the incremental benefit of including Ki67 in a multivariate prediction model for pCR. The first model did not take Ki67 into account as a predictive factor. In this model, cT, grading, ER, PR, and HER2 status were independent predictive factors for complete pathological remission (Table 2).

**Table 2:** Prediction of pathological complete remission without Ki67, using multiple linear regression analysis (final model)

Characteristic	OR	95% CI	*P*
pT			
1	1	-	-
2-4	0.39	0.21-0.73	< 0.01
Grading			
1-2	1	-	-
3	2.95	1.67-5.24	< 0.001
Estrogen receptor status			
Negative	1	-	-
Positive	0.28	0.13-0.57	< 0.001
Progesterone receptor status			
Negative	1	-	-
Positive	0.42	0.19-0.92	0.03
HER2/neu receptor status			
Negative	1	-	-
Positive	2.42	1.36-4.32	< 0.01

CI, confidence intervals; OR, odds ratio; pT, pathological tumor stage (TNM classification).

The second model included Ki67 in the logistic regression model. All of the parameters included in the first model continued to be significant predictive factors for pCR. In addition, Ki67 also proved to be an independent predictive factor for pCR, with an odds ratio of 3.51 (95% CI, 1.41 to 10.1; *P *= 0.01, Table 3). Using this model, the bootstrap-validated sensitivity and specificity rates were 82% and 75%, respectively. Using 20-fold cross-validation, these values represented 82% and 74%, respectively.

**Table 3:** Prediction of pathological complete remission with Ki67, using multiple linear regression analysis (extended final model)

Characteristic	OR	95% CI	P
pT			
1	1	-	-
2-4	0.34	0.18-0.65	< 0.01
Grading			
1-2	1	-	-
3	2.51	1.41-4.49	< 0.01
Estrogen receptor status			
Negative	1	-	-
Positive	0.30	0.14-0.63	< 0.01
Progesterone receptor status			
Negative	1	-	-
Positive	0.50	0.22-1.14	0.10
HER2/neu receptor status			
Negative	1	-	-
Positive	2.37	1.33-4.22	< 0.01
Ki67			
Low	1	-	-
High	3.51	1.41-10.10	0.01

CI, confidence intervals; OR, odds ratio; pT, pathological tumor stage (TNM classification).

### Comparison of the two prediction models

When the models were compared with each other using receiver operating characteristics, including Ki67 in the prediction model showed an improvement with regard to the area under the curve, although the benefit appeared to be marginal. The bootstrap-estimated AUC values were 0.83 without Ki67 and 0.84 with Ki67. The benefit appeared to be marginal because Ki67 status and pCR status are unbalanced (e.g., only seven patients with Ki67 low and without pCR) in such a way that the majority of the patients cannot be better classified by the extended model. The difference between the two models with regard to the likelihood ratio test was statistically significant (*P *< 0.01), although significance did not persist after bootstrap validation.

### Pathological remission rates in molecular subtypes

Table 4 shows the pCR rates for each category (triple-negative tumors, ER/PR-positive tumors, HER2-negative and HER2-positive tumors) in order to clarify the clinical value of Ki67 in absolute figures. There was a difference between tumors with high Ki67 proliferation and those with low proliferation for each molecular tumor subtype, but no statistical significance was found for triple negatives, as the number of triple-negative tumors with low proliferation was very small (seven of 122). Hormone receptor-positive tumors were associated with the lowest rates of pCR. Tumors that were ER-positive with a low Ki67 proliferation rate were associated with pCRs in 2.9% of cases, and tumors with a high Ki67 proliferation rate were associated with pCRs in 8.0% of cases (*P *= 0.03). One of the seven patients (14.3%) with triple-negative tumors with a low proliferation rate had a pCR; 60 of 122 patients with triple-negative tumors with high proliferation rates had pCRs (49.2%).

**Table 4:** Pathological complete response relative to molecular subtypes of tumor

	Tumor classification (+: yes/-: no)				Tumor response						
	
	ER/PR	HER2	Ki67	Neoadjuvant	All		pCR no		pCR yes		
					
				trastuzumab	N	%	n	%	n	%	*P*
Hormone receptor-positive	+	-	+	NA	176	100	162	92.0	14	8.0	0.05
	+	-	-	NA	140	100	136	97.1	4	2.9	
	+ or -	+	+	+ or -	88	100	49	55.7	39	44.3	0.03
	+ or -	+	-	+ or -	14	100	12	85.7	2	14.3	
HER2-positive	+ or -	+	+ or -	+	50	100	24	48.0	26	52.0	0.03
	+ or -	+	+ or -	-	52	100	37	71.2	15	28.8	
Triple negative	-	-	+	NA	122	100	62	50.8	60	49.2	0.12
	-	-	-	NA	7	100	6	85.7	1	14.3	
											

*All*	+ or -	+ or -	+ or -	+ or -	547	100	427	78.1	120	21.9	

ER, estrogen receptor; HER2, HER2/neu receptor; NA, not applicable; PR, progesterone receptor.

Approximately half of the HER2-positive patients received anti-HER2 treatment with trastuzumab in the neoadjuvant setting (n = 50), and 52% of these patients (n = 26) had pCRs. Patients who did not receive neoadjuvant trastuzumab only had pCRs in 29% of cases (n = 15). With regard to Ki67, the sample sizes were too small for comparison between the treated and untreated groups. When the two treatment groups were taken together, Ki67 was capable of distinguishing the HER2-positive group into one with a 14.3% pCR rate and one with a 44.3% pCR rate (*P *= 0.03) (Table 4).

### Empirical cut-off calculations

**Figure 2:**
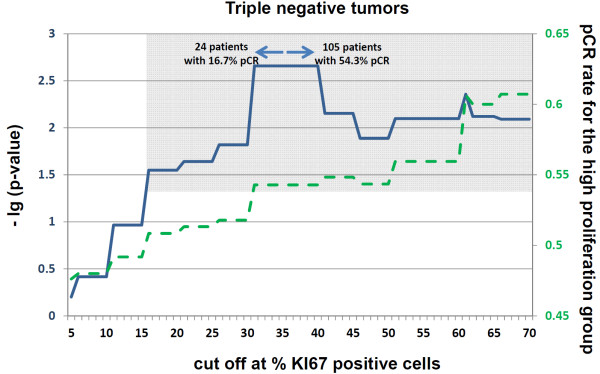
**Cut-off calculations for predicting a pathological complete response (pCR) in the group of triple-negative patients**. The continuous line represents the -lg of the *P *value for each respective cut-off point relative to the percentage of Ki67-positive stained tumor cells. The dashed line represents the proportion of patients with a pCR in the group of patients with the higher Ki67 values for each cut-off. The gray background indicates the area for which cut-offs with a significance level of 0.05 is reached. For the maximum of each -lg (*P *value), the sample size and pCR rates are provided in the figure.

**Figure 3:**
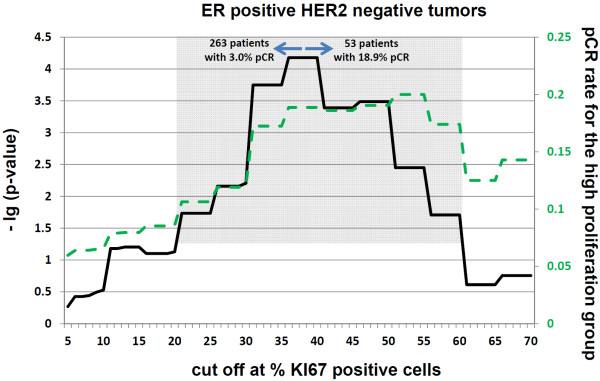
**Cut-off calculations for predicting a pathological complete response (pCR) in the group of ER/PR-positive and HER2-negative patients**. The continuous line represents the -lg of the *P *value for each respective cut-off point relative to the percentage of Ki67-positive stained tumor cells. The dashed line represents the proportion of patients with a pCR in the group of patients with the higher Ki67 values for each cut-off. The gray background indicates the area for which cut-offs with a significance level of 0.05 is reached. For the maximum of each -lg (*P *value), the sample size and pCR rates are provided in the figure.

**Figure 4:**
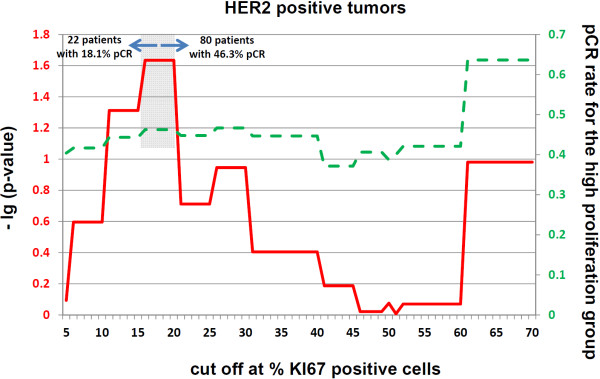
**Cut-off calculations for predicting a pathological complete response (pCR) in the group of HER2-positive patients**. The continuous line represents the -lg of the *P *value for each respective cut-off point relative to the percentage of Ki67-positive stained tumor cells. The dashed line represents the proportion of patients with a pCR in the group of patients with the higher Ki67 values for each cut-off. The gray background indicates the area for which cut-offs with a significance level of 0.05 is reached. For the maximum of each -lg (*P *value), the sample size and pCR rates are provided in the figure.

To improve the predictive value of Ki67 in each molecular subgroup, *P *values and pCR rates were calculated for each possible Ki67 value (Figure 2, 3, 4). In the triple-negative subgroup, this cut-off was between 30% and 40% positively stained cells. The corresponding odds ratio for this value was 5.9 (95% CI, 1.9 to 18.6; *P *= 0.002). With regard to the hormone receptor-positive, HER2-negative group, this Ki67 value was between 36% and 40% positively stained cells, with an OR of 7.4 (95% CI, 2.8 to 19.8; *P *< 0.0001). In the HER2-positive group, Ki67 as a predictive factor had the lowest *P *values at between 17% and 20% positively stained cells, with an OR of 3.9 (95% CI, 1.2 to 12.5; *P *= 0.02). None of these cut-off values were validated. For comparison of the effect of these cut-off values with a cut-off value of 13%, the respective pCR rates with these subgroup specific cut-off values are shown in Table 5.

**Table 5:** Pathological complete response relative to molecular subtypes of tumor with empirical cut-offs for Ki67

	Tumor classification (+: yes/-: no)				Tumor response						
	
	ER/PR	HER2	Ki67	Neoadjuvant	All		pCR no		pCR yes		
					
				trastuzumab	N	%	n	%	n	%	*P*
Hormone receptor-positive	+	-	≥ 38%	NA	53	100	43	81.1	10	18.9	< 0.0001
	+	-	< 38%	NA	263	100	255	97.0	8	3.0	
	+ or -	+	≥ 18%	+ or -	80	100	43	53.7	37	46.3	0.02
HER2-positive	+ or -	+	< 18%	+ or -	22	100	18	81.8	4	18.2	
	+ or -	+	+ or -	+	50	100	24	48.0	26	52.0	0.03
	+ or -	+	+ or -	-	52	100	37	71.2	15	28.8	
Triple negative	-	-	≥ 35%	NA	105	100	48	45.7	57	54.3	< 0.01
	-	-	< 35%	NA	24	100	20	83.3	4	16.7	
											

*All*	+ or -	+ or -	+ or -	+ or -	547	100	427	78.1	120	21.9	

ER, estrogen receptor; HER2, HER2/neu receptor; NA, not applicable; PR, progesterone receptor.

### Prognostic analysis of the cohort

As Ki67 did have an effect on the pCR, the additional value of pCR was tested with regard to the prognosis with multivariate Cox proportional hazard (PH) models for each of the outcome parameters -- overall survival (OS), distant disease-free survival (DDFS), and local recurrence-free survival (LRFS) -- firstly without the parameter of pCR, and then including it (Tables 6 and 7).

**Table 6:** Cox proportional hazard ratios without pCR variable for overall survival, distant disease-free survival, and local recurrence-free survival (three unique models)

Outcome	Characteristic		HR	95% CI	*P*
OS	PR	Negative	1	-	-
		Positive	0.62	0.39-0.99	0.05
	Ki67	Low	1	-	-
		High	7.07	2.82-17.75	< 0.0001
DDFS	cT	1	1	-	-
		2-4	2.69	0.98-7.37	0.05
	PR	Negative	1	-	-
		Positive	0.64	0.41-0.99	0.05
	Ki67	Low	1	-	-
		High	2.72	1.49-5.00	< 0.01
LRFS	PR	Negative	1	-	-
		Positive	0.49	0.24-1.02	0.06
	Ki67	Low	1	-	-
		High	1.76	0.74-4.21	0.21

CI, confidence intervals; cT, clinical tumor stage (TNM classification); DDFS, distant disease-free survival; HR, hazard ratio; LRFS, local recurrence-free survival; OS, overall survival; PR, progesterone receptor.

**Table 7:** Cox proportional hazard ratios including the pCR variable for overall survival, distant disease-free survival, and local recurrence-free survival (three unique models)

Outcome	Characteristic		HR	95% CI	*P*
OS	PR	Negative	1	-	-
		Positive	0.47	0.29-0.75	< 0.01
	Ki67	Low	1	-	-
		High	8.14	3.25-20.35	< 0.0001
	pCR	No	1	-	-
		Yes	0.18	0.07-0.45	< 0.001
DDFS	cT	1	1	-	-
		2-4	2.42	0.88-6.59	0.08
	PR	Negative	1	-	-
		Positive	0.49	0.32-0.76	< 0.01
	Ki67	Low	1	-	-
		High	3.24	1.77-5.89	< 0.001
	pCR	No	1	-	-
		Yes	0.16	0.07-0.43	< 0.001
LRFS	PR	Negative	1	-	-
		Positive	0.47	0.22-1.00	0.05
	Ki67	Low	1	-	-
		High	1.81	0.75-4.36	0.18
	pCR	No	1	-	-
		Yes	0.83	0.34-2.02	0.69

When pCR was not included, only PR positivity and Ki67 had prognostic value for OS and DDFS. The clinical tumor stage (cT) also had prognostic value relative to DDFS. None of the parameters showed clear statistical significance relative to LRFS (Table 6). When pCR was included, Ki67 maintained a statistically significant prognostic value for OS and DDFS, but not for LRFS (Table 7). Including pCR improved the prognostic value of the model, with differences in the chi-squared value (likelihood ratio test) of 21.2 for OS and 23.8 for DDFS (both *P *< 0.00001). Bootstrap validation reduced these values only slightly (data not shown).

**Figure 5:**
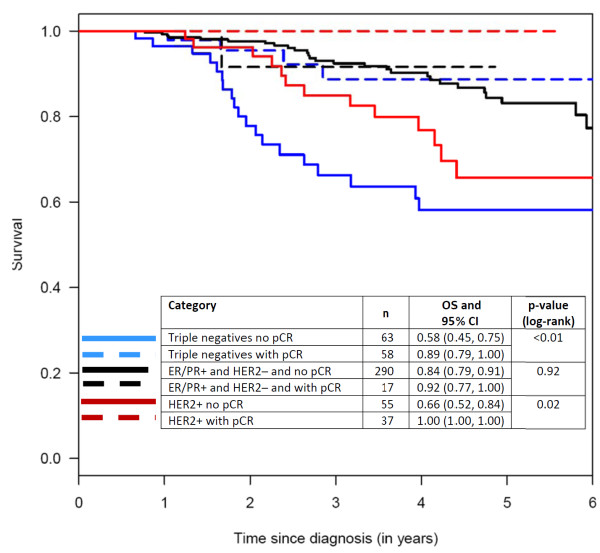
**Kaplan-Meier curves with 5 year survival rate estimates (OS) and respective 95% confidence intervals (CI) for overall survival according to pathological complete responses (pCRs)**. ER, estrogen receptor; HER2, HER2/neu receptor; PR, progesterone receptor.

**Figure 6:**
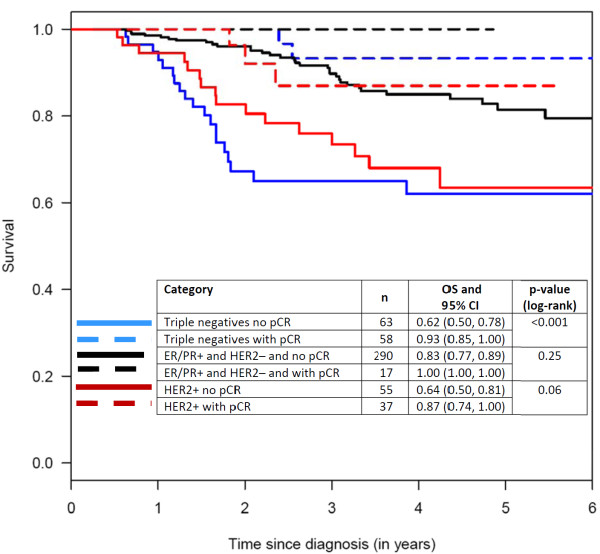
**Kaplan-Meier curves with 5 year survival rate estimates (OS) and respective 95% confidence intervals (CI) for distant disease-free survival according to pathological complete responses (pCRs)**. ER, estrogen receptor; HER2, HER2/neu receptor; PR, progesterone receptor.

**Figure 7:**
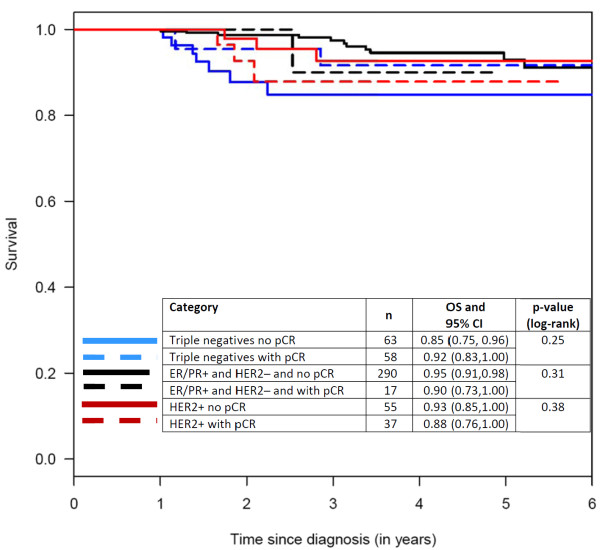
**Kaplan-Meier curves with 5 year survival rate estimates (OS) and respective 95% confidence intervals (CI) for **local recurrence-free survival **according to pathological complete responses (pCRs)**. ER, estrogen receptor; HER2, HER2/neu receptor; PR, progesterone receptor.

To display these effects, Kaplan-Meier curves were constructed for the following groups of patients: those with triple-negative tumors, with and without pCR; those with HER2-positive tumors, with and without pCR; and those with ER/PR-positive and HER2-negative tumors, with and without pCR (Figure 5, 6, 7). Patients in the pCR groups had better outcomes than the corresponding patients without pCRs. This effect was statistically significant relative to DDFS for patients with triple-negative tumors and those with ER/PR-positive tumors, and also relative to OS for patients with triple-negative tumors and HER2-positive tumors.

**Figure 8:**
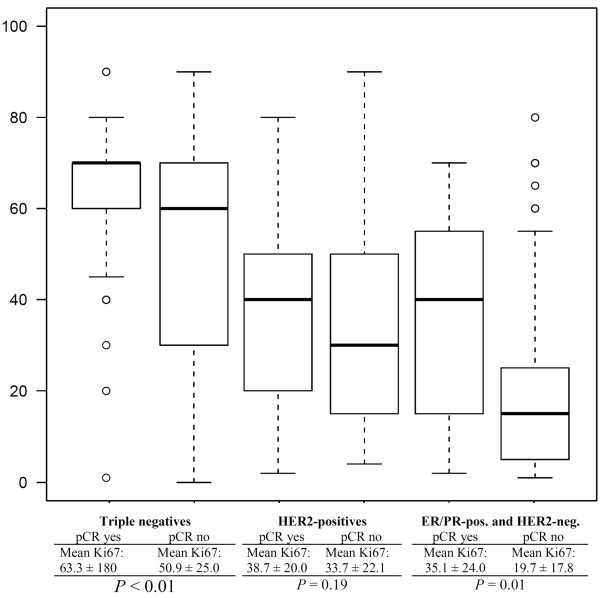
**Box plots for Ki67 as a continuous variable (0-100% positive staining of assessed cells) in the different groups relative to estrogen receptor status (ER), progesterone receptor status (PR), HER2/neu receptor status, and pathological complete remission (pCR)**.

An analysis of the prognosis in different molecular subgroups, different Ki67 groups, and different pCR behaviors did not seem reasonable due to very small sample sizes in some of the groups. Mean and median Ki67 values were therefore calculated for each of the groups of Kaplan-Meier estimates from the continuous Ki67 variable, to give an impression of the Ki67 value for each of the Kaplan-Meier curves. Box plots, means, and *P *values for these values are shown in Figure 8.

## Discussion

This retrospective study investigated the value of Ki67 as a predictive factor in relation to neoadjuvant chemotherapy and possible effects on prognosis. Ki67 was found to be an independent predictor for pathological complete responses and for the prognosis in all patients across all subtypes. Looking at Ki67 values in different molecular subtypes, it seemed that patients with *triple-negative *or *hormone receptor-positive, HER2-negative *breast cancer had a more favorable prognosis when a pCR was achieved, although these patient groups had a higher Ki67 proliferation rate. These results might suggest that the Ki67 cut-off values in patients undergoing chemotherapy may need to be set at a higher level in these subgroups to allow prediction of the chemotherapy response with a translation to the prognosis.

In the present study, the pCR rate in patients with triple-negative tumors (47.3%) was in the range reported in previously published studies (22-58%) [8,16-19]. The pCR rates in HER2-positive carcinomas (28% without and 52% with neoadjuvant trastuzumab treatment) were higher than in the NOAH study (19% and 38%) [20], GeparQuattro study (31.7% for patients treated with trastuzumab) [21], and TECHNO study (42% for patients treated with trastuzumab) [22], but the sample sizes in the present study were much smaller. The pCR rate in the HER2-negative, hormone receptor-positive group (5.7%) was also consistent with that in other published studies [23]. The present study also confirms previous reports that pCR is associated with a more favorable prognosis in some molecular subgroups [8,24].

Previously published parameters such as age, BMI, tumor stage, histological type, hormone receptor and HER2 status correlated with pCR, as in other previously published studies [23,25]. Ki67 also had a strong correlation with pCR and added independently to the predictive value of a logistic regression model. This effect was present with regard to the total group of patients and with regard to the molecular subtypes of breast cancer as defined by hormone receptor and HER2 status. It reached statistical significance in the hormone and HER2 receptor-positive groups, with a cut-off at 13%, but not with regard to the triple negative group. Cut-off calculations within the molecular subgroups showed that much better differentiation between treatment response groups could be achieved with much higher Ki67 cut-off values for the hormone receptor-positive group (between 36% and 40%) and the triple-negative group (between 30% and 40%). For the HER2-positive group, the cut-off value was between 17% and 20%. However, this group is difficult to interpret, as it included patients with and without neoadjuvant trastuzumab treatment.

Triple-negative tumors generally have a much higher proportion of Ki67-positive cells, and differentiation between responsiveness groups could thus be expected at a higher level. However, in the hormone receptor-positive group, previous molecular analysis determined a cut-off at 13% to differentiate between luminal A and luminal B tumors [10]. This cut-off does not appear to be the best for predicting the chemotherapy response.

Patients with tumors that have a very high level of proliferation might possibly have a better prognosis than those with lower Ki67 values as a result of a successful therapy response. In the present study, this was shown indirectly for the triple-negative and hormone receptor--positive subgroup. Patients with a pCR had a better prognosis and a higher mean Ki67 value, whereas patients with a lower Ki67 value had a more unfavorable prognosis. This might explain some of the inconsistencies in reports concerning the prognostic value of Ki67 [5].

One aspect of the present study involves both advantages and disadvantages. On the one hand, the methods of Ki67 staining and evaluation used are part of routine clinical practice. The staining and assessment of whole sections may be a strength, as most published studies use tissue microarrays and are unable to account for heterogeneously expressed Ki67 in a whole slide section. In addition, the fixation and staining procedures were carried out directly after the fixation and embedding of the core biopsies into paraffin. This may have reduced the potential for variability in studies using paraffin blocks of different ages, ranging up to decades. On the other hand, routine clinical assessment means that different batches of chemicals and antibodies are used, and also that there are different observers involved. Another problematic issue might concern the arbitrary molecular classification of tumors used [26]. Approximately 30% of luminal B tumors are HER2-positive [27]. Patients with these tumors were included in the HER2-positive group in the present study. However, further subcategorization was not possible due to the small sample sizes in the subgroups, and not all of the HER2-positive patients were treated with trastuzumab.

It would have been desirable to validate other cut-off values for Ki67 in a sample set of patients treated with chemotherapy, but the sample size in the present study appeared to be too small to pursue this aim.

## Conclusions

Ki67 provides additional and independent predictive information regarding the response to chemotherapy and the prognosis in a group of patients receiving neoadjuvant treatment for breast cancer. It would be easy to include it in the panel of markers routinely assessed in clinical practice. The findings of the present study suggest that this marker could help select patients who are unable to benefit from chemotherapy, such as those with HER2-negative and hormone receptor-positive tumors with low proliferation. On the other hand, however, it is more difficult to identify patients who are definitely capable of benefiting from chemotherapy with a response, and it is also difficult to translate response results into a general prognosis. Further research is needed in groups of patients undergoing chemotherapy that are large enough for these issues to be addressed in relation to the distinct molecular subtypes of breast cancer and in which two cut-off values for the prognosis can be taken into account.

## Competing interests

The authors declare that they have no competing interests.

## Authors' contributions

All authors contributed meaningfully to this manuscript, in particular: PAF participated in statistical analyses and drafted the manuscript; KH, MN, AH, CMB, CR, RSW, MRB, MS, LK, MPL, and MWB were involved in obtaining patient and tumor characteristics and carrying out surgical procedures; LH performed statistical analyses; JDS, AH, AD, and DLW were involved in histopathological and immunohistochemical analyses. All authors have read and approved the final manuscript.

## Pre-publication history

The pre-publication history for this paper can be accessed here:

http://www.biomedcentral.com/1471-2407/11/486/prepub
